# Prevalence and resistance to antibacterial agents in *Salmonella enterica* strains isolated from poultry products in Northern Kazakhstan

**DOI:** 10.14202/vetworld.2023.657-667

**Published:** 2023-03-27

**Authors:** Anara Mendybayeva, Zulkyya Abilova, Aitbay Bulashev, Raushan Rychshanova

**Affiliations:** 1 Research Institute of Applied Biotechnology, A. Baitursynov Kostanay Regional University, Kostanay, Kazakhstan; 2 Department of Veterinary Medicine, A. Baitursynov Kostanay Regional University, Kostanay, Kazakhstan; 3 Department of Microbiology and Biotechnology, S. Seifullin Kazakh Agrotechnical University, Astana, Kazakhstan

**Keywords:** antibiotic resistance, food safety, poultry, resistance genes, *Salmonella*

## Abstract

**Background and Aim:**

*Salmonella* is one of the main causative agents of foodborne infections. The source of the pathogen, in most cases, is poultry products. The intensification of poultry farming and the constant and uncontrolled use of antimicrobials has led to an increase in the level of antibiotic resistance, especially in developing countries. This study aimed to determine the level of sensitivity to antimicrobial agents in *Salmonella enterica* strains isolated from poultry products in Northern Kazakhstan, as well as to determine the genetic mechanisms of resistance and the presence of integrons.

**Materials and Methods:**

In total, 398 samples of poultry products sold in Northern Kazakhstan were selected. *Salmonella* strains were isolated from product samples using microbiological methods. *Salmonella* was identified based on morphological, biochemical, and serological methods, as well as polymerase chain reaction (PCR). Sensitivity testing for antimicrobial agents was performed using the disk diffusion method. The detection of resistance genes was performed using PCR and gel electrophoresis.

**Results:**

Out of 398 samples of poultry products, a total of 46 *Salmonella* isolates were obtained. Most of the isolates belong to the serovar *Salmonella* Enteritidis (80.4%). The assessment of sensitivity to antibacterial agents showed that *Salmonella* was mainly resistant to nalidixic acid (63%), furadonin (60.9%), ofloxacin (45.6%), and tetracycline (39.1%). In 64.3% of cases, *Salmonella* was resistant to three or more groups of antibacterial agents. Resistance genes such as *tet*A, *tet*B, *bla*TEM, *aad*A, *sul*3, and *cat*II, as well as integrons of two classes (*teg*1 and *teg*2), were identified.

**Conclusion:**

Poultry products contain antimicrobial-resistant strains of *Salmonella*, as well as genes encoding resistance mechanisms. The results emphasize the need for constant monitoring of not only pathogenic microorganisms but also their sensitivity to antimicrobial agents. The potential threat to human health requires a unified approach to the problem of antibiotic resistance from representatives of both public health and the agroindustrial complex.

## Introduction

According to a Food and Agriculture Organization (FAO) report, chicken meat is the most popular type of meat globally, possibly due to its low price. Chicken meat accounts for 40.6% of meat production worldwide [1]. According to this report, the projected global consumption of poultry meat by 2030 will be 151.83 metric tons (in slaughter weight) [2]. According to the Bureau of National Statistics [3], as of February 1, 2022, the poultry population in Kazakhstan equaled 47,313 thousand heads, of which 8935.4 thousand heads were kept in Northern Kazakhstan. Moreover, approximately 369.7 million eggs are produced, of which 114.2 million are produced in Northern Kazakhstan per year. Food safety is directly related to food pathogens such as *Salmonella*. According to Callejón *et al*. [4], salmonellosis, along with campylobacteriosis, is the most common foodborne disease globally and is believed to spread mainly through chicken meat. The damage from diseases and cost of combating zoonosis are significant.

Asymptomatic bacterial transmission of *Salmonella* is often noted in the poultry population, which is the main factor in spreading bacteria through poultry products [5]. According to Antunes *et al*. [6], there is an epidemiological relationship between the prevalence of salmonellosis in humans and consumed poultry products. According to the Unified Sanitary and Epidemiological and Hygienic Requirements for Products [7] approved by the Decision of the Customs Union Commission, the presence of *Salmonella* in 25 g of livestock products, as well as in 125 g of eggs, is not allowed.

*Salmonella* circulating in animal products, in particular, poultry products are the main source of human food infections [6]. To date, ~2600 *Salmonella* serotypes have been identified, of which most pathogenic *Salmonella* serotypes belong to *Salmonella enterica* [8]. The use of antimicrobials for intensive animal husbandry and poultry farming has led to the natural selection of pathogenic microorganisms resistant to many antimicrobials [9]. The emergence and spread of resistant strains of *Salmonella* to a wide range of antibacterial agents have led to an increase in severe cases of diseases and mortality. Thus, by 2050, the number of deaths associated with resistance to antibacterial agents is expected to reach 10 million [10]. In recent years, an increase in the number of *Salmonella* with resistance to multiple antibacterial agents in poultry products has been reported [11]. For example, *Salmonella* isolated in China from broiler chicken showed resistance to medicines such as piperacillin (96.1%), ampicillin (AMP) (95.3%), and nitrofurantoin (96.31%) [12], and 75% of these showed resistance to multiple agents. In Korea, a high level of resistance to nalidixic acid (NA) (78.5%) and gentamicin (GEN) (52.3%) by *Salmonella* isolated from chicken was reported [13].

The rapid spread of antibiotic-resistant strains of *Salmonella* is a growing public health problem worldwide [14]. In recent years, the number of cases with *Salmonella* resistance to clinically significant antimicrobials, such as fluoroquinolones and third-generation cephalosporins, has increased [15, 16]. A serious problem of recent decades, not only for human medicine [17] but also for animals [18], is the appearance of strains producing extended-spectrum beta-lactamases that are capable of hydrolyzing beta-lactam antibiotics.

In addition, resistance genes and integrons contribute to the spread of *Salmonella* resistance to antibacterial agents [19]. Resistance to sulfamethoxazole/trimethoprim (SMX-TMP), beta-lactams, and tetracyclines (TETs) is due to the presence of resistance genes such as *sul*1, *dfr*A, *bla*TEM, and *tet*A [20]. In turn, integrons play an important role in the acquisition and spread of resistance to multiple medications in both *Salmonella* and other Gram-negative bacteria because they capture resistance gene cassettes [21]. Studies have shown that integrons contribute not only to the spread of antimicrobial resistance genes, but also accelerate the development of multiple resistance [22–24].

This study aimed to determine the level of sensitivity to antimicrobial agents in *S. enterica* strains isolated from poultry products in Northern Kazakhstan, as well as the genetic mechanisms of resistance and the presence of integrons.

## Materials and Methods

### Ethical approval

Live animals and birds, as well as other wildlife objects, were not used in this study. Therefore, obtaining permission from the ethics commission was not required. Samples of poultry products were purchased from retail outlets.

### Study period and location

The study was conducted from November 2019 to December 2021 in the Laboratories of Microbiology and Molecular Genetic Analysis of the Research Institute of Applied Biotechnology of the A. Baitursynov Kostanay Regional University.

### Bacterial isolates

In this study, we used *Salmonella* strains isolated from November 2019 to December 2021 in Northern Kazakhstan. The source of *Salmonella* isolates was poultry products and products containing chilled or frozen chicken meat. In total, 398 samples of chicken meat (n = 138), eggs (n = 130), and semi-finished products (n = 130) were selected from retail outlets, markets, and supermarkets. All the studied samples were produced in Kazakhstan. The selected samples were delivered to the laboratory on the same day for further study. The process of sampling, isolation, and identification of samples was repeated every 2 weeks from November 2019 to December 2021.

The isolation and identification of *Salmonella* were performed according to the guidelines of the Federal Service for Supervision of Consumer Rights Protection and Human Welfare [25]. Briefly, 25 g of each sample was homogenized with 225 mL of buffered peptone water and incubated at 37°C for 18–24 h. Then, they were transplanted to selective enrichment media: Rappaport-Vassiliadis medium (HiMedia Laboratories, India) and Muller-Kaufman medium (HiMedia Laboratories). After the enrichment stage, seeding was performed on differential diagnostic media, such as bismuth-sulfite agar (BSA) (Merck, Germany) and xylose-lysine-deoxycholate agar (Merck), as well as on the CHROMagar *Salmonella* chromogenic medium (CHROMagar, France) and incubated at 37°C for 18–24 h (BSA; 48 h). After the incubation process, *Salmonella*-typical colonies were seeded on nutrient agar and Kligler medium (HiMedia Laboratories). Identification was performed by Gram staining to determine tinctorial characteristics. We performed the following tests of enzymatic characteristics: Growth on media with citrates (State Scientific Center for Applied Microbiology and Biotechnology, Russia), presence of lysine decarboxylase (Biotechnovatsiya LLC, Russia), phenylalanine deaminase (Biocompas-S LLC, Moscow, Russia), ability to decompose urea (Biocompas-S LLC, Russia), methyl red test, Voges–Proskauer test (Pharmacotherapy Research Center CJSC, Russia), and indole test (HiMedia Laboratories Pvt. Ltd.). In addition, we used a commercial test system (ENTEROtest24, ErbaLachema, Czech Republic). Serotyping was performed by agglutination on a slide using O- and H-antigenic sera (Petsal, Russia). The interpretation was performed according to the Kauffman-White classification scheme [26].

### Testing of sensitivity to antibacterial agents

Testing was performed using the Kirby–Bauer disk diffusion method according to European Committee on Antimicrobial Susceptibility Testing (EUCAST) recommendations [27]. The following disks with antibacterial agents were used in work: β-lactams (AMP 10 μg, amoxicillin: 25 μg, cefoperazone [CPR]: 75 μg, cefoxitin [CFN]: 30 μg, cefpodoxime [CFM]: 10 μg), aminoglycosides (streptomycin [STR]: 10 μg, kanamycin [KAN]: 30 μg, GEN: 120 μg), amphenicols (levomycetin: 30 μg), TESs (TET: 30 μg, doxycycline [DOX]: 30 μg), fluoroquinolones (enrofloxacin [ENR]: 5 μg, ciprofloxacin: 5 μg, norfloxacin: 10 μg, ofloxacin [OF]: 5 μg), quinolones (NA: 30 μg), sulfonamides (sulfamethoxazole/trimethoprim: 1.25/23.75), and nitrofurans (furadonin [FD]: 300 μg, furazolidone: 300 μg) (FBUN Pasteur Research Institute of Epidemiology and Microbiology, Russia). The interpretation of the test results was performed according to EUCAST recommendations [28]. The data of the test results were interpreted as stable, intermediate, and sensitive. The index of multiple antibiotic resistance (MAR) for resistant strains of *Salmonella* was calculated as described [29].

### DNA isolation

*Salmonella* strains resistant to antibacterial agents were inoculated on the surface of meat-peptone agar and incubated at 37°C for 24 h. The complete loop of the daily culture was dissolved in water without DNase and heated at 100°C for 10 min, sharply cooled on ice, and centrifuged at 10,000× g [30]. After centrifugation, the supernatant was selected and used for polymerase chain reaction (PCR) testing.

### Molecular characterization of isolates

*Salmonella* isolates were identified by 16S rRNA sequencing. The reaction mixture in a volume of 20 mL consisted of 10 μL of master mix (DreamTaq Green PCR Master mix 2×, Thermo Fisher Scientific, Waltham, Massachusetts, USA), 1 μL of forward 27F (5’-AGAGTTTGATYMTGGCTCAG-3’), reverse 515R (5’-TTACCGCGGCKGCTGGCAC-3’) universal primer [31], 1 μL of DNA, and 7 μL of water without nucleases. Amplification was performed at 55°C on a Proflex thermal cycler (Applied Biosystems, USA). Amplification products were visualized by electrophoresis in 1.5% agarose gel (Quantum gel documenting system, Vilber, Collégien, France). The sequencing of bacterial 16S rRNA gene fragments was performed using the Big Dye Terminator v3.1 Cycle Sequencing Kit according to the manufacturer’s protocol (BigDye^®^ Terminator v3.1 Cycle Sequencing Kit Protocol, Applied Biosystems). The sequencing results were processed using SeqA. The search for homologous nucleotide sequences of the 16S rRNA genes was performed using the BLAST program in the International Gene Bank database of the US National Center for Biotechnology Information (NCBI). Phylogenetic analysis was performed using the MEGA6 software (https://www.megasoftware.net/). Nucleotide sequence alignment was performed using the ClustalW algorithm version 1.6 (http://www.clustal.org/).

### Determination of resistance genes

Testing was performed by multiplex PCR with detection in agarose gel. The reaction mixture in a volume of 20 μL consisted of forward and reverse primers (Synthol, Moscow, Russia), DreamTaq Green PCR Master mix 2× (ThermoFisher Scientific), water without nucleases (ThermoFisher Scientific), and DNA. Primers and amplification modes (Table-1) [32–52] were used as described by Rychshanova *et al*. [53]. Amplification products were visualized by electrophoresis in 1.5% agarose gel (QUANTUM gel documenting system).

**Table-1:** Primers used to detect Salmonella antibiotic resistance genes.

Group of proteins causing resistance	Gene	Primer	Sequence (5’- 3’)	Class of antibiotics and AMP	Reference
Dihydropteroate synthase	*sul*1-F	*sul*1-F	CTTCGATGAGAGCCGGCGGC	Sulfonamides	[32]
		*sul*1-R	GCAAGGCGGAAACCCCGCC		
	*sul*2-F	*sul*2-F	GCGCTCAAGGCAGATGGCATT		[33]
		*sul*2-R	GCGTTTGATACCGGCACCCGT		
	*sul*3-F	*sul*3-F	CATTCTAGAAAACAGTCGTAGTTCG		[34]
		*sul*3-R	CATCTGCAGCTAACCTAGGGCTTTGGA		
Dihydrofolate reductase	*dfr*1	*dfr*1-F	ACGGATCCTGGCTGTTGGTTGGACGC	Trimethoprim	[35]
		*dfr*1-R	CGGAATTCACCTTCCGGCTCGATGTC		
	*dfr*5	*dfr*5-F	GCBAAAGGDGARCAGCT	Trimethoprim	[36]
		*dfr*5-R	TTTMCCAYATTTGATAGC		
	*dfr*A7	*dfr*A7-F	AAAATTTCATTGATTTCTGCA		[36]
		*dfr*A7-R	TTAGCCTTTTTTCCAAATCT		
Chloramphenicol acetyltransferase Chloramphenicol carriers	*cat*II	*cat*II-F	ACACTTTGCCCTTTATCGTC	Amphenicols	[36]
		*cat*II-R	TGAAAGCCATCACATACTGC		
	*cml*A	*cml*A-F	TTGCAACAGTACGTGACAT		[37]
		*cml*A-R	ACACAACGTGTACAACCAG		
Beta-lactamases	*tem*	*bla*TEM-F	ATCAGTTGGGTGCACGAGTG	Beta lactams	[38]
		*bla*TEM-R	ACGCTCACCGGCTCCAGA		
	*shv*	*bla*SHV-F	CGCCGGGTTATTCTTATTTGTCGC		[39]
		*bla*SHV-R	TCTTTCCGATGCCGCCGCCAGTCA		
	*oxa* 1	*OXA* I-F	ATGAAAAACACAATACATATCAAC		[40]
		*OXA* I-R	AAAGGACATTCACGCCTGTG		
	*ctx* M	CTX-MF	TTTGCGATGTGCAGTACCAGTAA		[41]
		CTX-MR	CCGCTGCCGGTCTTATC		
		CTX-M2F	ATGATGACTCAGAGCATTCGCCGC		[42]
		CTX-M2R	TCAGAAACCGTGGGTTACGATTTT		
Tetracycline carriers	*tet*A	*tet*A-F	GCTACATCCTGCTTGCCT	TETs	[43]
		*tet*A-R	CATAGATCGCCGTGAAGA		
	*tet*B	*tet* (B)-F	CATTAATAGGCGCATCGCTG		[44]
		*tet* (B)-R	TGAAGGTCATCGATAGCAGG		
Aminoglycoside phosphotransferases	*aphA1*	*aph*A1-F	AAACGTCTTGCTCGAGGC	Aminoglycosides	[32]
		*aph*A1-R	CAAACCGTTATTCATTCGTGA		
Aminoglycoside acetyltransferases	*aacA4*	*aac*A4-F	ATGACTGAGCATGACCTTGCG		[45]
		*aac*A4-R	TTAGGCATCACTGCGTGTTCG		
	aac (3) II	aac (3) II-F	ACTGTGATGGGATACGCGTC		[46]
		aac (3) II-R	CTCCGTCAGCGTTTCAGCTA		
Adenosyltransferase aminoglycosides	*aad*B	*aad*B-F	ATGGACACAACGCAGGTCGC		[45]
		*aad*B-R	TTAGGCCGCATATCGCGACC		
	*aad*A	*aad*A-F	GTGGATGGCGGCCTGAAGCC		[47]
		*aad*A-R	ATTGCCCAGTCGGCAGCG		
DNA gyrases and topoisomerases IV	*qnr*A	*qnr*A-F	ATTTCTCACGCCAGGATTTG	Quinolones	[48]
		*qnr*A-R	GATCGGCAAAGGTTAGGTCA		
	*qnr*B	*qnr*B-F	GATCGTGAAAGCCAGAAAGG		[49]
		*qnr*B-R	ACGATGCCTGGTAGTTGTCC		
	*qnr*5	*qnr*5-F	ACGACATTCGTCAACTGCAA		
		*qnr*5-R	TAAATTGGCACCCTGTAGGC		
	*qep*A	*qep*A-F	GCAGGTCCAGCAGCGGGTAG		[50]
		*qep*A-R	CTTCCTGCCCGAGTATCGTG		
Streptomycin phosphotransferases	*str*A	*str*A-F	CCAATCGCAGATAGAAGGC	Streptomycins	[51]
		*str*A-R	CTTGGTGATAACGGCAATTC		
	*str*B	*str*B-F	GGATCGTAGAACATATTGGC		
		*str*B-R	ATC GTC AAG GGA TTG AAA CC		
In*teg*rons	*Teg*	*Teg*1-F	CAGTGGACATAAGCCTGTTC		[52]
		*Teg*1-R	CCCGAGGCATAGACTGTA		
		*Teg*2-F	TTATTGCTGGGATTAGGC		
		*Teg*2-R	ACGGCTACCCTCTGTTATC		

AMP=Ampicillin

### Statistical analysis

Microsoft Excel 2017 was used for data analysis. The index of MAR was determined for each isolate by the formula MAR = a/b, where a is the number of antibiotics to which the tested isolate demonstrates resistance and b is the total number of antibiotics to which sensitivity testing was performed.

## Results

A total of 46 *Salmonella* isolates (11.5%) were obtained from 398 poultry samples. Morphological and cultural characteristics of the obtained isolates were characteristic of *Salmonella* spp.

**Figure-1:**
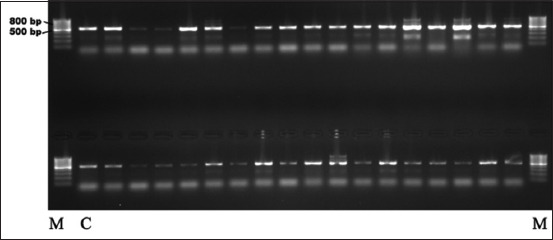
Electrophoregram of 16S rRNA. M=Marker, C=Control sample.

The obtained isolates corresponded to their subspecies and serotypes according to their biochemical characteristics. The antigenic characteristics of *Salmonella* isolates showed the presence of seven serotypes, including *Salmonella* Enteritidis (80.4%, 37/46), *Salmonella* Paratyphi C (6.5%, 3/46), *Salmonella* Typhimurium (4.3%, 2/46), *Salmonella* Blegdam (2.2%, 1/46), *Salmonella* Tennessee (2.2%, 1/46), *Salmonella* Moscow (2.2%, 1/46), and *Salmonella* Dublin (2.2%, 1/46). Molecular characterization of *Salmonella* isolates using 16S rRNA showed 100% compliance (Figure-1). The length of the amplification product was 525 bp.

The results of phylogenetic analysis of 16S rRNA gene sequences are presented as phylogenetic trees, where the level of homology with *Salmonella* strains from the NCBI database ranged from 98% to 100%.

The evaluation of sensitivity to antibacterial agents showed that 91.3% (42/46) of *Salmonella* isolates were resistant to at least one agent (Table-2).

**Table 2:** Index of resistance to multiple antibacterial agents, phenotypes, and genotypes of *Salmonella* resistance.

No.	*Salmonella* serovar	Selection source	Resistance phenotype	MAR index	Number of antimicrobial resistance (AMR) groups	Genotypic resistance	Integrons
1	*S.* Blegdam	Chicken eggs	OF, NA, FRN, FD	0.21	3		
2	*S.* Dublin	Minced chicken	TET, ENR, CIP, OF, NA, FD	0.31	4		
3	*S.* Moscow	Chicken meat	OF, NA, FRN, FD	0.16	3		
4	*S.* Tennessee	Chicken dumplings	AMP, AMX, CPR, CFN, KAN, LEV, TET, DOX, ENR, NOR, OF, NA, SMX-TMP, FRN, FD	0.79	8	*ctx*M, *aphA1*, *aad*A, *tet*A, *sul*3, *cml*A, *cat*II	*teg*1, *teg*2
5	*S.* Typhimurium	Chicken fillet	FD	0.05	1		
6	*S.* Paratyphi C	Minced chicken	CFN, TET, DOX, ENR, CIP, NOR, OF, NA, FRN, FD	0.53	5	*aad*A, *tet*A, *qnr*B	*teg*1
7	*S.* Paratyphi C	Minced chicken	CFN, LEV, TET, DOX, CIP, NOR, OF, NA, SMX-TMP, FRN, FD	0.58	7	*tet*A, *sul*3, *cml*A, *cat*II	*teg*1
8	*S.* Paratyphi C	Mixed minced meat (beef+chicken)	AMP, AMX, CFN, STR, LEV, TET, DOX, CIP, NOR, OF, NA, SMX-TMP, FRN, FD	0.74	8	*aad*B, *tet*A, *tet*B, *sul*3, *cat*II, *qnr*A	*teg*1
9	*S.* Enteritidis	Chicken liver	FRN, FD	0.1	1		
10	*S.* Enteritidis	Chicken eggs	TET, DOX	0.1	1		
11	*S.* Enteritidis	Chicken legs	FD	0.05	1		
12	*S.* Enteritidis	Halal dumplings	ENR, NA	0.1	2		
13	*S.* Enteritidis	Chicken fillet	ENR, OF, NA	0.16	2		
14	*S.* Enteritidis	Chicken breast	FD	0.05	1		
15	*S.* Enteritidis	Chicken eggs	OF, NA, FRN, FD	0.16	3		
16	*S.* Enteritidis	Chicken carcass	OF, NA, FRN, FD	0.16	3		
17	*S.* Enteritidis	Khinkali	OF, NA, FRN, FD	0.16	3		
18	*S.* Enteritidis	Broiler chicken carcass	OF, NA, FRN, FD	0.16	3		
19	*S.* Enteritidis	Dumplings	CFN, LEV, TET, DOX	0.21	3		
20	*S.* Enteritidis	Chicken breast	OF, NA, FRN, FD	0.16	3		
21	*S.* Enteritidis	Chicken eggs	OF, NA, FRN, FD	0.16	3		
22	*S.* Enteritidis	Chicken eggs	CFN, TET, NA, FRN, FD	0.26	4	*tet*B	-
23	*S.* Enteritidis	Chicken carcass	AMP, AMX	0.1	1		
24	*S.* Enteritidis	Soup set, chicken	OF, NA, FRN, FD	0.16	3		
25	*S.* Enteritidis	Minced chicken	CFN, LEV, TET, DOX	0.31	4	*bla*TEM, *aacA4*	-
26	*S.* Enteritidis	Chicken eggs	TET, ENR, OF, NA, FRN, FD	0.31	4		
27	*S.* Enteritidis	Chicken thighs	AMP, AMX, SMX-TMP, FD	0.21	3		
28	*S.* Enteritidis	Chicken eggs	CPR, STR, TET, OF, FRN, FD	0.31	5		
29	*S.* Enteritidis	Chicken eggs	CFN, TET, ENR, NA, FRN, FD	0.31	5		
30	*S.* Enteritidis	Sausages	OF, NA, FRN, FD	0.16	3		
31	*S.* Enteritidis	Dumplings	LEV, DOX, NOR, OF, NA, FRN, FD	0.37	5		
32	*S.* Enteritidis	Chicken drumsticks	NA	0.05	1		
33	*S.* Enteritidis	Chicken fillet	TET, DOX, ENR, OF, NA, SMX-TMP, FRN, FD	0.42	5	*bla*TEM, *aad*A, *dfr*1	-
34	*S.* Enteritidis	Broiler chicken carcass	STR, TET, DOX, ENR, CIP, NOR, OF, NA, SMX-TMP	0.47	5	*tet*A, *sul*2, *dfr*1, *str*A, *str*B	-
35	*S.* Enteritidis	Broiler chicken	AMP, AMX, TET	0.16	2	-	*teg*1
36	*S.* Enteritidis	Broiler chicken carcass	AMP, AMX, TET, DOX, ENR, SMX-TMP	0.31	4	*bla*TEM, *OXA*1, *sul*2, *qep*A	-
37	*S*. Enteritidis	Broiler chicken carcass	TET, DOX	0.1	1	*tet*B	-
38	*S*. Enteritidis	Broiler chicken carcass	OF, NA, FRN, FD	0.1	2		
39	*S*. Enteritidis	Broiler chicken fillet	TET, DOX, ENR, CIP, OF, NA, SMX-TMP, FRN, FD	0.47	5	*bla*TEM, *OXA*1, *tet*A	-
40	*S*. Enteritidis	Chicken fillet	CIP, OF, NA	0.16	2	*tet*A	-
41	*S*. Enteritidis	Halal dumplings	CIP, NA	0.1	2		
42	*S*. Enteritidis	Halal khinkali	CIP, NA	0.1	2		

R is resistant, I is intermediate, and *S* is sensitive. FD=Furadonin, FRN=Furazolidone, *SM*X-TMP=*Su*lfameth*oxa*zole/ trimethoprim, NA=Nalidixic acid, OF=Ofl*oxa*cin, NOR=Norfl*oxa*cin, CIP=Ciprofl*oxa*cin, ENR=Enrofl*oxa*cin, DOX=Doxycycline, TET=Tetracycline, LEV=Levomycetin, GEN=Gentamicin, KAN=Kanamycin, *ST*R=*St*reptomycin, CFM=Cefpodoxime, CFN=Cefoxitin, CPR=Cefoperazone, AMX=Amoxicillin, AMP=Ampicillin. *S.* Enteritidis=*Salmonella* Enteritidis, *S.* Paratyphi C=*Salmonella* Paratyphi C, *S.* Typhimurium=*Salmonella* Typhimurium, *S.* Tennessee=*Salmonella* Tennessee, *S.* Moscow=*Salmonella* Moscow, *S.* Dublin=*Salmonella* Dublin, *S.* Blegdam=*Salmonella* Blegdam

**Figure-2:**
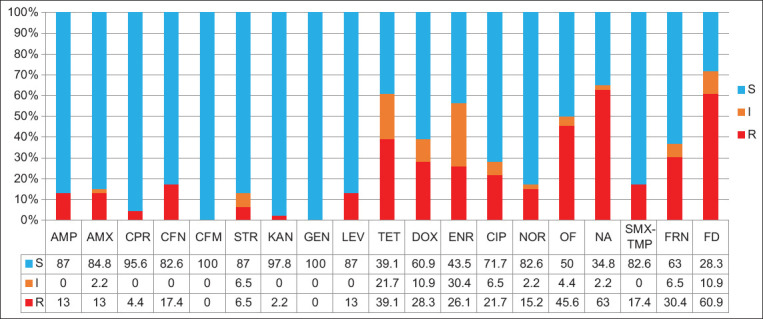
Resistance to antibacterial agents in *Salmonella*. R is resistant, I is intermediate, and S is sensitive. FD=Furadonin, FRN=Furazolidone, SMX-TMP=Sulfamethoxazole/trimethoprim, NA=Nalidixic acid, OF=Ofloxacin, NOR=Norfloxacin, CIP=Ciprofloxacin, ENR=Enrofloxacin, DOX=Doxycycline, TET=Tetracycline, LEV=Levomycetin, GEN=Gentamicin, KAN=Kanamycin, STR=Streptomycin, CFM=Cefpodoxime, CFN=Cefoxitin, CPR=Cefoperazone, AMX=Amoxicillin, AMP=Ampicillin.

In this study, only four strains (8.7%) showed sensitivity to all tested antibacterial agents (Table-2). Most of the isolated strains showed resistance to NA (63%), FD (60.9%), OF (45.6%), and TET (39.1%) (Figure-2). Resistance to the group of beta-lactams and aminoglycosides was less frequently observed. Of the beta-lactam group, the largest number of strains were resistant to CFN (17.4%), and of the aminoglycosides, to STR (6.5%). A high level of intermediate resistance to ENR (30.4%) and TET (21.7%) was noted. All *Salmonella* isolates studied were sensitive to CFM and GEN.

In the context of pharmacological groups, antimicrobial resistance ranged from 30.4% to 60.9% for nitrofurans, 15.2%–63% for fluoroquinolones, 28.3%–39.1% for TETs, 2.2%–6.5% for aminoglycosides, and 4.4%–17.4% for beta-lactams. *Salmonella* isolates had 33 resistance models (Table-2). Only four resistance profiles were repeated, namely, OF-NA-FD (6 times), CIP-NA (2 times), TET-DOX (2 times), and FD (3 times).

Two isolates of *Salmonella*, namely, *S*. Tennessee (Table-2, no. 4) and *S*. Paratyphi C (Table-2, no. 8), showed resistance to all eight tested groups of antibacterial agents at once. The index of multiple resistances of these isolates was as follows: *S*. Tennessee: 0.79, *S*. Paratyphi C: 0.74. One strain was resistant to seven antibiotic groups at once (*S*. Paratyphi C, Table-2, no. 7), and the multiple resistance index of the isolate was 0.58. Isolates resistant to three groups of antibiotics at once (12/42) were the most common. Isolates resistant to five (7/42) or four (5/42) groups were less often observed. Isolates resistant to three or more groups of antibacterial agents were classified as multi-resistant strains. This group included 27 of 42 strains (64.3%). Thus, the predominant number of *Salmonella* strains showed resistance to pharmacological groups such as nitrofurans, quinolones, and TETs.

Testing for the presence of genotypic resistance of *Salmonella* from 42 antibiotic-resistant isolates revealed 13 strains (13/42, 30.9%) carrying genes encoding resistance to antibacterial agents (Table-2).

A total of 42 phenotypically resistant strains of *Salmonella* showed 19 resistance genes and integrons of two classes (Table-2). Thus, in 16.6% of cases, the *tet*A gene encoding the efflux pump of TET was detected. The *bla*TEM gene was detected in 9.5% of the cases. The *aad*A, *sul*3, *cat*II, and *tet*B genes were detected in 7.1% of cases. The genes *cml*A, *dfr*1, *sul*2, and *OXA*1 were detected in 4.7% of cases. The remaining genes were identified in individual cases.

The discovery of Class 1 integrons responsible for the horizontal transfer of resistance genes is noteworthy. All three strains of *S*. Paratyphi C isolated in this study showed a high level of resistance to antibacterial agents. These isolates were resistant to 5–8 pharmacological groups of medications at once and were classified as extremely resistant forms. In addition, the isolates of *S*. Paratyphi C carried genes directly to three or more groups of antibiotics. In another extremely resistant strain of *S*. Tennessee resistant to eight groups of medications at once, nine resistance genes were found, including integrons of two classes. Resistance genes were most often detected in chicken meat and only in one case, in chicken eggs.

## Discussion

The prevalence of *Salmonella* in poultry products isolated in Northern Kazakhstan was 11.5%. The data obtained were lower than the results of studies conducted in China [54] at 23% or in Egypt at 28.6% [20]. Nevertheless, the level of contamination in this study was higher than that in Brazil, where it equals 3.6% [11]. The low prevalence of *Salmonella* in Brazil is explained by the introduction of good manufacturing practices and hazard analysis and critical control points. In Spain, where it equals 7.8% [55], the low levels of *Salmonella* excretion may be associated with the absence of an enrichment procedure at the stage of bacterial isolation. In Russia, where it equals 10.7% [56], the low prevalence of *Salmonella* is associated with a decrease in the number of foci of *Salmonella* infection. The presence of *Salmonella* in poultry products highlights the potential risk of infection to humans.

The antigenic characteristics of the isolated *Salmonella* strains showed that *S*. Enteritidis (80.4%) was the predominant serotype. Consistently, in China [12], *S*. Enteritidis was the predominant serotype (40.3%) isolated from chicken carcasses; in Iran [57], *S*. Enteritidis was the predominant serotype in 43% of cases of contamination of poultry carcasses; and in India [58], *S*. Enteritidis was the predominant serotype (68.1%) in poultry products. The percentage of the prevalence of *S*. Enteritidis in this study was higher than similar data obtained from Saudi Arabia (40%) [59] and Pakistan (9%) [60]. The infectious nature of [58, 61] *S*. Enteritidis and its ability to colonize the gastrointestinal tract and reproductive organs of birds led to the widespread distribution of this serotype in chicken meat. According to Herikstad *et al*. [62], the serotype *S*. Enteritidis is the predominant serotype detected in poultry products, particularly in chicken meat.

The *Salmonella* strains isolated in this study showed a high level of resistance to antibiotics, such as NA (63%), FD (60.9%), OF (45.6%), and TET (39.1%). Similar data on antibiotic resistance have been recorded in countries such as Russia [56], with a significant number (40.6%) of TET-resistant strains; Korea, with a high level NA-resistant strains (85%) [63]; Romania, with 54.9% of TET-resistant strains and 43.1% NA-resistant strains [64]; and China, with 99.5% NA-resistant and 51.9% TET-resistant strains [14] of *Salmonella*. Compared to other studies of *Salmonella* isolated from chicken carcasses in Brazil, resistance to sulfonamides (75%–100%) was predominant, whereas resistance to NA and nitrofurans was absent [11]. A low level of resistance was registered to CPR (4.4%), KAN (2.2%), and STR (6.5%). All the tested isolates were sensitive to GEN and CFM.

The high level of antimicrobial resistance may be primarily due to their uncontrolled use as growth stimulants, as well as for the treatment and prevention of infections. In Kazakhstan, the veterinary and sanitary regulations do not restrict the use of antimicrobials both for therapeutic (prophylactic) purposes and as growth promoters. In addition, it is recommended to use antibiotics for prophylactic purposes in animals without clinical manifestations located in farming centers unfavorable for listeriosis. As a preventive measure, it is recommended to use antibiotics and feeds enriched with antibiotics for fish in rubella-affected areas. The only restriction on the use of antibiotics is mandatory registration in the Republic of Kazakhstan [65]. One of the ways of spreading resistance to microorganisms is the lack of a management system and disposal of residual amounts of medications [66].

This study has shown resistance to all tested groups of antibacterial agents. However, high resistance to medications such as NA, ENR, and OF is of particular concern. Resistance to these substances indicates a potential decrease in sensitivity to the group of fluoroquinolones belonging to critically important antimicrobial agents, according to the WHO list [67]. The identified phenotypes of *Salmonella* resistance showed that isolated strains were resistant more often to nitrofurans, TETs, and fluoroquinolones and less often to beta-lactams and aminoglycosides. Similar resistance phenotypes have been recorded in Shandong Province, China [14]. However, the data obtained differed from the results obtained in Egypt [68], where resistance to sulfonamides prevailed in 100% of cases; Great Britain [69], where *Salmonella* isolates were resistant to TET (26.27%), sulfonamides (23.72%), and AMP (21.43%), and Malaysia [70], where all the studied *Salmonella* strains were resistant to penicillin but sensitive to TET.

In this study, *Salmonella* isolates showed multiple resistances in 64.3% of cases. The isolates had 33 resistance models, which is more than that in China, where 25 resistance models were found [14].

Among the isolates of *Salmonella*, 19 genes of resistance to antibacterial agents, as well as integrons of two classes were identified. Studies in Asian countries, such as Bangladesh [22] and China [14], have shown that TET resistance in most cases is due to the presence of the *tet*A gene. Our studies have also shown the presence of the *tet*A gene in isolates resistant to the TET group. In addition to Class A TET resistance genes, Class B genes responsible for encoding efflux pumps were found. The main mediators of resistance to beta-lactam antibiotics have been identified, namely, the *bla*TEM genes [13]. Resistance to sulfonamide preparations was due to the acquisition of *sul*2 and *sul*3 genes, encoding dihydropteroate synthase.

In this study, isolated cases of plasmid-mediated fluoroquinolone resistance genes (*qnr*A and *qnr*B) were detected [71]. Integrons of classes 1 and 2 were found to be associated with horizontal gene transfer, as well as with the spread of resistance to multiple medications among Enterobacteriaceae [72].

The high frequency of detection of antibiotic-resistant *Salmonella* isolates from poultry products can become a serious problem for both veterinary medicine and public health. Although heat treatment of meat products and eggs can reduce infection [73], the risk of transmission of resistance genes to the human gut microbiota remains.

## Conclusion

This study showed the presence of *Salmonella* strains in poultry products sold in retail outlets in Northern Kazakhstan. The predominant *Salmonella* serotype was the *S*. Enteritidis serotype. Although the prevalence of *Salmonella* in poultry products is relatively low (11.5%), there is a potential risk to public health. Our findings revealed the presence of resistance to multiple medications in *Salmonella* strains. In addition, genes encoding resistance mechanisms, such as enzymatic modification, changes in the permeability of cell membranes, and changes in the structure of the target, were identified. The data obtained confirm the need to introduce and maintain strict hygiene and sanitation standards at all stages of poultry carcass processing, as well as in the production of semi-finished products. The introduction of practices, such as hazard analysis and critical control points and good manufacturing practices, will help reduce the incidence of *Salmonella* contamination. Continuous monitoring of antimicrobial resistance and reasonable use of medications in poultry farming are necessary to reduce the emergence and spread of resistance to multiple medications in zoonotic pathogens.

## Data Availability

The supplementary data can be available from the corresponding author on a reasonable request.

## Authors’ Contributions

AB and RR: Conceptualization and design of the study and participated in the writing of the manuscript. ZA: Carried out data acquisition and participated in the writing of the manuscript. AM: Carried out analysis and interpretation of data and participated in the writing of the manuscript. All authors have read, reviewed, and approved the final manuscript.
